# Expression Level of HIV-1 Vif Can Be Fluctuated by Natural Nucleotide Variations in the *vif*-Coding and Regulatory SA1D2prox Sequences of the Proviral Genome

**DOI:** 10.3389/fmicb.2019.02758

**Published:** 2019-11-28

**Authors:** Naoya Doi, Takaaki Koma, Akio Adachi, Masako Nomaguchi

**Affiliations:** ^1^Department of Microbiology, Tokushima University Graduate School of Medical Science, Tokushima, Japan; ^2^Department of Microbiology, Kansai Medical University, Osaka, Japan

**Keywords:** HIV-1, *vif*, SA1D2prox, natural variation, expression level, subtype

## Abstract

Vif is required for HIV-1 replication in natural target cells by counteracting host restriction factors, APOBEC3 (A3) proteins. We recently demonstrated that Vif expression level can be changed by naturally occurring single-nucleotide variations within SA1D2prox of the HIV-1 genome. We also found that levels for *vif*/*vpr* mRNAs are inversely correlated. While amino acid sequence per se is critical for functionality, Vif expression level modulated by signal sequences in its coding region is likely to be important as well. There are two splicing sites in the region involved in *vpr* expression. To reveal possible fluctuations of Vif-expression level, we examined SA1D2prox and *vif* gene by chimeric approaches using HIV-1 subtypes B and C with distinct anti-A3 activity. In this report, recombinant clones in subtype B backbone carrying chimeric sequences with respect to SA1D2prox/*vif* and those within the *vif*-coding region were generated. Of these, clones containing *vif*-coding sequence of subtype C, especially its 3′ region, expressed *vif*/Vif at a decreased level but did at an increased level for *vpr*/Vpr. Clones with reduced *vif*/Vif level grew similarly or slightly better than a parental clone in weakly A3G-positive cells but more poorly in highly A3G-expressing cells. Three clones with this property were also tested for their A3-degrading activity. One of the clones appeared to have some defect in addition to the poor ability to express *vif/*Vif. Taken all together, our results show that natural variations in the SA1D2prox and *vif*-coding region can change the Vif-expression level and affect the HIV-1 replication potential.

## Introduction

HIV-1 Vif antagonize host intrinsic restriction factors, A3 proteins (A3s) (Malim and Emerman, 2008; Harris et al., 2012; Malim and Bieniasz, 2012; Aydin et al., 2014; Desimmie et al., 2014; Feng et al., 2014; Okada and Iwatani, 2016). A3 family of cytidine deaminases consists of seven members (A, B, C, D, F, G, and H). Of these, A3D, A3F, A3G, and certain haplotypes of A3H potently inhibit HIV-1 replication, A3G in particular, by deaminase-dependent and -independent mechanisms. HIV-1 Vif inactivates A3s through proteasomal degradation by recruiting them to E3 ubiquitin ligase complexes (Hultquist et al., 2011; Refsland et al., 2012, 2014; Ooms et al., 2013; Aydin et al., 2014; Desimmie et al., 2014; Feng et al., 2014; Salter et al., 2014; Okada and Iwatani, 2016). Proper counteraction of Vif against A3s is necessary for optimal HIV-1 replication.

HIV-1 Vif is highly divergent in patients’ samples. Several natural variations in Vif negatively affect its anti-A3 activity (Simon et al., 2005; De Maio et al., 2011; Ooms et al., 2013; Peng et al., 2013; Refsland et al., 2014). Vifs derived from different HIV-1 subtypes also counteract A3s in a varying degree. Even among isolates from the same subtype, the ability of Vif to overcome A3 restriction has been shown to be different (Iwabu et al., 2010; Binka et al., 2012; Lisovsky et al., 2013). Difference in Vif amino acid sequence can thus alter anti-A3 activity, thereby influencing HIV-1 replication ability and the hypermutation rate of viral genome introduced by A3 deaminase.

**Figure 1:**
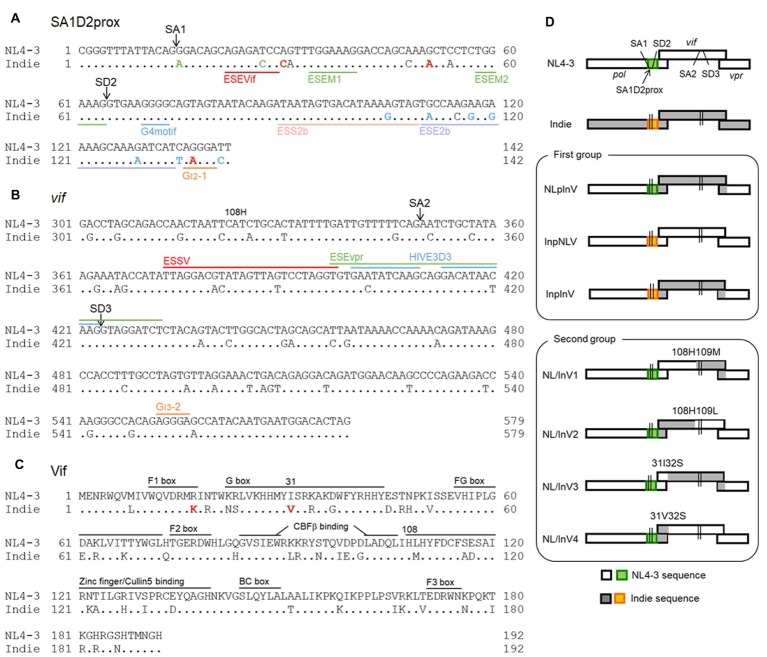
Sequence alignment and genome organization around the *vif* gene of HIV-1 proviral clones used in this study. Proviral clones of HIV-1 subtype B (NL4-3, HIV-1_NL4-3_; GenBank: AF324493) (Adachi et al., 1986) and subtype C (Indie, HIV-1_IndieC1_; GenBank: AB023804) (Mochizuki et al., 1999) were used in this study. Dots in the nucleotide and amino acid sequences of Indie show the nucleotides and residues identical to those of NL4-3. Sequence identities between regions of NL4-3 and Indie are as follows. (1) Nucleotide identity. SA1D2prox, 89%. *Vif*: nos. 1 to 324 (amino acid no. 108), 87%; no. 325 to 3′end, 87%; nos. 1 to 93 (amino acid no. 31), 91%; no. 94 to 3′end, 86%. (2) Amino acid identity for Vif. Nos.1 to 108, 78%; no. 109 to C-terminus, 78%; nos. 1 to 31, 80%; no. 32 to C-terminus, 78%. **(A)** Nucleotide sequence alignment of SA1D2prox region. SA1 and SD2 sites are indicated. Based on our previous results (Nomaguchi et al., 2014, 2016), single-nucleotide variations within the SA1D2prox in NL4-3 that decrease and increase *vif* production levels are indicated by green and red letters, respectively, in the Indie sequence. Single-nucleotide variations for which their effects on the *vif* production are not much or have not been determined yet are represented as blue and black letters, respectively, in the Indie sequence. Reported SREs, i.e., ESEVif (Exline et al., 2008), ESEM1/M2 (Kammler et al., 2006), G4 motif (Exline et al., 2008), ESS2b (Brillen et al., 2017), ESE2b (Brillen et al., 2017), and G_I2_-1 (Widera et al., 2013) are indicated. See also a review (Sertznig et al., 2018). **(B)** Alignment of the *vif*-coding sequence (positions from 301 to 579). SA2, SD3, and amino acid residue H at position 108 of the Vif protein are indicated. Known splicing silencers, ESSV (Madsen and Stoltzfus, 2005), HIVE3D3 (Tsuruno et al., 2011), and G_I3_-2 (Widera et al., 2014), and a splicing enhancer, ESEvpr (Erkelenz et al., 2013) are shown as reference. **(C)** Alignment of Vif amino acid sequence. Red letters indicate residues (17 K and 31 V) reported to be responsible for high anti-A3G activity of Vif derived from HIV-1 subtype C (Iwabu et al., 2010). Numbers 31 and 108 marked above sequences show the amino acid positions that were utilized to generate chimeric Vif. Domains that are important for proteasomal degradation of A3s are indicated for reference (Feng et al., 2014; Nakashima et al., 2015). **(D)** Genome organization around the *vif* gene of the HIV-1 proviral clones constructed in this study. SA1D2prox regions derived from NL4-3 and Indie are shown in green and orange, respectively. SA1, SD2, SA2, and SD3 are indicated in all clones as shown. Above the chimeric *vif* gene, the corresponding amino acid residues at positions 31/32 and 108/109 of Vif are indicated. Recombinant viral clones between NL4-3 and Indie were generated by amplifying chimeric regions with overlapping PCR as indicated at amino acid positions and then by introducing resultant PCR fragments into NL4-3 using unique sites (*Sbf*I in *pol* and *Eco*RI in *vpr*).

HIV-1 generates ~50 mRNAs species coding the nine viral proteins through alternative splicing using splicing donors (SD1–SD4) and splicing acceptors (SA1–SA7) (Purcell and Martin, 1993; Amendt et al., 1995). HIV-1 mRNA production is highly regulated process, and *vif* mRNA is generated by utilizing SD1 and SA1. Several splicing regulatory elements (SREs) in the HIV-1 genome and numerous host proteins are involved in the process (Caputi, 2011; Karn and Stoltzfus, 2012; Sertznig et al., 2018). We previously demonstrated that *vif* mRNA/Vif protein expression levels are altered by naturally occurring single nucleotide variations (nSNVs), found within the region around SA1/SD2 through investigation of the HIV-1 sequence compendium^1^. The region was then named as SA1D2prox (Figure 1A). We also observed the inverse correlation between levels of *vif*/*vpr* mRNAs and the Vif/A3G-dependent virus growth fluctuation (Widera et al., 2014; Nomaguchi et al., 2016). Moreover, we found that the RNA stem-loop structure formed in the region containing SA1 (Watts et al., 2009; Pollom et al., 2013) can contribute to determination of *vif* mRNA production level (Nomaguchi et al., 2017). On the one hand, sequence of *vif*-coding region also contains SA2/SD3 involved in *vpr* mRNA creation, and various SREs close to SA2/SD3 sites have been reported (Figure 1B; Karn and Stoltzfus, 2012; Sertznig et al., 2018). Considering the mutually related *vif*/*vpr* levels and the presence of important elements for splicing, we hypothesized that the levels of *vif*/*vpr* and thus, those of Vif/Vpr, may be changeable by sequence variations of the *vif*-coding region.

In this work, to confirm and extend our previous findings described above, we examined the sequences of SA1D2prox and *vif* from HIV-1 subtypes B and C. It has been reported that the subtype C virus shows a higher anti-A3G activity than the subtype B, and that the amino acids responsible for the difference were determined (Iwabu et al., 2010). To link this finding to our previous results, we generated chimeric viruses between the two subtypes that exhibit distinct anti-A3G activity. Here, we have summarized the results obtained for the chimeric viruses, and proposed that viral nucleotide sequence of the *vif*-coding region is also important for *vif*/Vif expression, in addition to the SA1D2prox regulatory sequence.

## Comparison of Nucleotide/Amino Acid Sequences Around the *vif* Genes of NL4-3 (HIV-1 Subtype B) and Indie (HIV-1 Subtype C) Virus Clones

Based on analysis of the HIV-1 sequence compendium^1^, we have shown that nSNVs found within SA1D2prox (142 nucleotide-length region from Pol-Integrase R224cgg to just before *vif* start codon) can alter Vif expression level/growth potential of HIV-1_NL4-3_ (Figure 1A; Nomaguchi et al., 2014, 2016). We were interested in the difference in nucleotide sequence that may affect *vif*/*vpr* expression levels of subtypes B (NL4-3 clone) and C (Indie clone) viruses. First, SA1D2prox sequence of Indie clone from HIV-1 subtype C was compared to that of NL4-3 clone from subtype B. Nucleotide sequences of this region between two clones were different (89% sequence identity), and several variations that increase or decrease *vif* production level were present in SA1D2prox region in the Indie genome (Figure 1A). Second, we compared *vif*-coding sequence between the two clones. This region contains SA2 and SD3 in addition to various splicing enhancer (ESEvpr) (Figure 1B; Erkelenz et al., 2013) and splicing silencers (ESSV, HIVE3D3, and G_I3_-2) (Figure 1B; Bilodeau et al., 2001; Tsuruno et al., 2011; Widera et al., 2014). Comparison of the entire *vif* gene between NL4-3 and Indie exhibited 87% sequence identity. Nucleotide difference in the *vif*-coding sequence of the two clones was also observed in the region around SA2/SD3 and in various SREs (Figure 1B).

Various functional and interacting domains in Vif, which are required for interaction with A3s and subsequent proteasomal degradation, have been identified (Figure 1C; Aydin et al., 2014; Desimmie et al., 2014; Feng et al., 2014; Salter et al., 2014). Identity of amino acid sequence between NL4-3 and Indie Vifs is 78%, and differences were present in the domains important for anti-A3 activity. These differences are likely to affect viral anti-A3 activity. Of note, the Vif sequence of Indie contains 17 K and 31 V amino acid residues that are associated with high anti-A3G activity of Vif derived from subtype C (Iwabu et al., 2010). In total, it is quite possible that sequence differences in SA1D2prox and *vif* gene of the subtype B NL4-3 and subtype C Indie may influence their anti-A3 activity through different *vif*/*vpr* expression levels. Previous studies (Widera et al., 2013; Brillen et al., 2017; Sertznig et al., 2018) strongly support this prediction.

## Generation of Proviral Recombinant Clones With Chimeric Sequences of SA1D2prox and *vif* Gene

In most studies on antagonism of Vif and A3s reported to date (Aydin et al., 2014; Desimmie et al., 2014; Feng et al., 2014; Salter et al., 2014), the anti-A3 activity of various Vif proteins and/or the restriction activity of various A3s was evaluated by using expression vectors, not proviral clones. However, several elements in the HIV-1 genome, such as SA1D2prox (Nomaguchi et al., 2016), can alter Vif expression level. Furthermore, in the alternative splicing of HIV-1 genome composed of the sequential multi-event process, long-range interactions between splicing sites occur (Pollom et al., 2013; Emery et al., 2017). Thus, we investigated, in the context of proviral genome, effects of nucleotide sequence variations in the SA1D2prox and *vif* regions on the *vif*/*vpr* expression levels and on the HIV-1 growth potential. Figure 1D shows recombinant viral clones generated in this study as test proviral clones in an NL4-3 backbone. We did not construct chimeric clones with a backbone of Indie, because the clone can grow in peripheral blood mononuclear cells (Jere et al., 2004) but not in a CXCR4/CCR5-positive cell line MT4/CCR5 (Nomaguchi et al., 2013a). Recombinant clones thus generated were classified into two groups (Figure 1D): (1) clones that have chimeric sequences with respect to the SA1D2prox and *vif* gene (NLpInV, InpNLV, and InpInV) and (2) clones that have chimeric sequences at amino acid positions 31 or 108 within Vif (NL/InV1, NL/InV2, NL/InV3, and NL/InV4).

## Alterations in the Expression Levels of *vif*/*vpr* and Vif/Vpr Observed for Chimeric Proviral Clones

**Figure 2:**
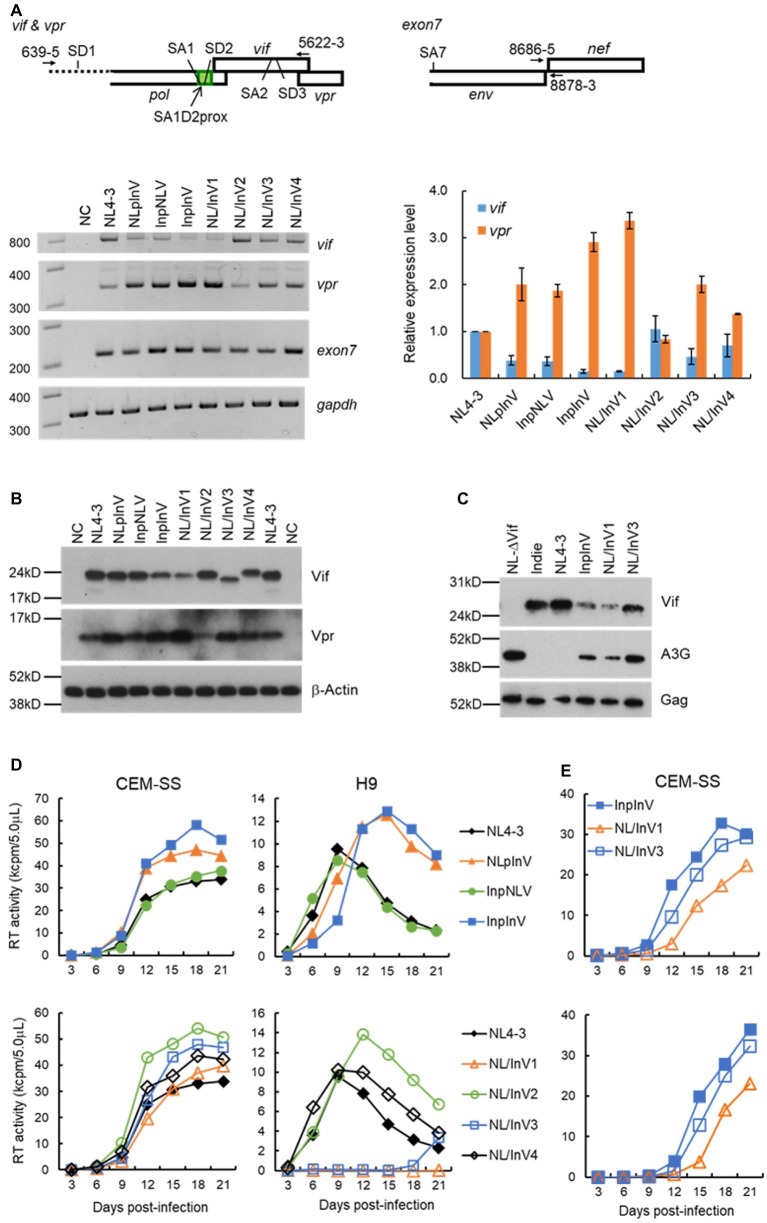
Virological characteristics of NL4-3 and chimeric viral clones newly constructed. Most experiments here were performed similarly as described previously (Nomaguchi et al., 2014, 2016, 2017). **(A)** Semiquantitative PCR analysis. Indicated proviral clones (2.5 μg) were transfected into 293T cells by Lipofectamine 2000 (Thermo Fischer Scientific), and cell lysates were made at 18 to 20 h post-transfection. Total RNAs were prepared and subjected to cDNA synthesis with oligo(dT) primer. *Vif*/*vpr* mRNAs were amplified simultaneously in one reaction using the cDNA as template and a specific primer pair indicated at the top. The reverse primer 5,622-3 was designed for the 100% matched sequence between NL4-3 and Indie. *Exon7* (amplified by a primer pair indicated at the top) and *gapdh* were used as a transfection control (total level of HIV-1 mRNAs) and an internal control, respectively. Representative data from three independent experiments are presented in the lower left portion of this panel. *Vif* and *vpr* mRNA levels relative to those of NL4-3 are presented in the lower right portion of this panel. Expression levels of *vif* and *vpr* mRNAs in each sample were normalized by those of all HIV-1 mRNAs (*exon7*) and *gapdh*. Mean values ± standard errors from three independent experiments are shown. **(B)** Western blotting analysis. 293T cells were transfected with 3.5 μg of proviral clones indicated by Lipofectamine 2000, and on day 1 post-transfection, cell lysates were prepared. To detect Vif and Vpr proteins, the polyclonal anti-Vif peptide antibody (Akari et al., 1999) and anti-Vpr peptide antibody (#3951, NIH Research and References Reagent Program) were used, respectively. These rabbit polyclonal antibodies were raised against a synthetic NL4-3 Vif peptide (amino acids 170-184; amino acid identity between NL4-3 and Indie is 9/15 = 60%) and against a synthetic NL4-3 Vpr peptide (amino acids 1-46; amino acid identity between NL4-3 and our chimeric clones NLpInV, InpInV, NL/InV1, and NL/InV3 is 45/46 = 98%). β-actin was used as an internal control. Representative data from at least two independent experiments are shown. **(C)** Comparative analysis of the A3G-degrading activity. Ability of virus clones to degrade A3G in cells was assessed by the co-transfection experiment as previously described (Yamashita et al., 2008, 2010). A flag-tagged A3G expression vector (0.1 μg) and a proviral clone (2.5 μg) were co-transfected into 293T cells, and the A3G expression level in the cells at 48 h post-transfection was monitored by Western blotting analysis as described in **(B)**. For detection of the flag-tagged A3G and control Gag (precursor p55) proteins, anti-Flag (Sigma) and anti-Gag (#3537, NIH Research and References Reagent Program) antibodies were used, respectively. As a negative control, NL-Nd (∆Vif) which lacks the Vif expression (Adachi et al., 1991) was used. Representative data from two independent experiments are shown. **(D)** Growth kinetics in CEM-SS and H9 cells. Viruses were prepared from 293T cells transfected with indicated proviral clones (2.5–5.0 μg) by Lipofectamine 2000 or calcium-phosphate co-precipitation method, and virus amounts were determined by the virion-associated reverse transcriptase (RT) assays (Willey et al., 1988; Nomaguchi et al., 2013b). Equal amounts of viruses (10^4^ RT units) were inoculated into a weakly A3G-positive cell line CEM-SS and a highly A3G-expressing cell line H9 (10^5^ cells). Culture supernatants were collected every 3 days, and virus replication was monitored by RT assays. All viruses were examined for their growth properties in the same single experiment. Results obtained for chimeric clones were separately presented in the upper and lower portions of this panel for clarity, and the same NL4-3 data were shown in both graphs for easy comparison. Representative data from at least three independent infection experiments performed using virus samples prepared by separate transfections are shown. **(E)** Growth kinetics in CEM-SS cells. A series of infection experiments were performed as described in the legend to **(D)**, and results from two independent experiments for InpInV, NL/InV1, and NL/InV3, other than those shown in **(D)**, are presented at upper and lower panels.

We have previously showed that nSNVs within SA1D2prox of HIV-1_NL4-3_ genome can increase or decrease the expression level of *vif*/Vif (Nomaguchi et al., 2016) and that they concomitantly alter the *vpr* level in an inverse correlation (Widera et al., 2014; Nomaguchi et al., 2016). Here, we analyzed effects of sequence variations in SA1D2prox and *vif* on the *vif*/*vpr* mRNA production by semiquantitative PCR as previously described (Nomaguchi et al., 2016). As shown in Figure 2A, *vif* production levels of NLpInV, InpNLV, and InpInV (the first group of chimeric viruses in Figure 1D as described above) were clearly reduced relative to that of NL4-3, indicating that *vif* level is decreased by Indie SA1D2prox and *vif* sequences. In contrast, the second group of virus clones carrying chimeric *vif* (NL/InV1, NL/InV2, NL/InV3, and NL/InV4 in Figure 1D) gave different results. While the *vif* level of NL/InV2 and NL/InV4 was comparable to that of NL4-3, NL/InV1, and NL/InV3, especially NL/InV1, exhibited a considerable reduction in *vif* level (Figure 2A). This reduction may be due to the Indie nucleotide sequence containing SA2/SD3 in *vif* (Figure 1D). Consistent with previous reports (Widera et al., 2014; Nomaguchi et al., 2016), *vpr* levels of chimeric viral clones were found to inversely correlate with *vif* levels (Figure 2A). Moreover, in good agreement with our data here, another study of the HIV-1 splicing based on detailed next-generation sequencing analyses has revealed that the usage of SD1-SA2 (Figure 2A), critical for producing *vpr* mRNA, clearly occupies a higher proportion in the total transcripts of subtype C (clone pZM247Fv2) than for subtype B (clone NL4-3) (Emery et al., 2017). InpInV and NL/InV1 with conspicuous decrease in *vif* expression showed remarkable increase in *vpr* production, suggesting that the splice sites involved in *vif*/*vpr* mRNA production are mutually exclusive as previously described (Widera et al., 2014; Nomaguchi et al., 2016).

To confirm above results (Figure 2A) by the protein expression, we then performed Western blot analysis of 293T cells transfected with chimeric clones and monitored Vif/Vpr expression levels. As shown in Figure 2B, the anti-Vif antibody could recognize all Vif proteins examined and gave results fairly in parallel with those for *vif* level. The results obtained showed that NLpInV, InpNLV, and NL/InV2 expressed Vif at a level comparable to NL4-3, and that InpInV, NL/InV1, NL/InV3, and NL/InV4 produced Vif at a lower level relative to NL4-3, the former three in particular. Observed Vif level for NLpInV and InpNLV was apparently higher than that expected from the *vif* expression level (Figure 2A). This could be due to the assay systems used in this study. The semiquantitative PCR analysis may be more sensitive than the Western blot analysis to detect differences between samples. As for Vpr expression levels, results obtained appeared to be more variable among viral clones tested than those for Vif levels (Figure 2B). Compared with NL4-3, clones producing a relatively decreased level of Vif (InpInV, NL/InV1, and NL/InV3) expressed a relatively high level of Vpr. Vpr level relative to that of NL4-3 were increased for NLpInV but slightly for InpNLV and NL/InV4. Only NL/InV2 exhibited a lower level of Vpr relative to NL4-3. Difference in Vpr level among clones tested was more obvious than that noted in *vpr* level: e.g., NLpInV vs. InpNLV, InpInV vs. NL/InV1, and NL4-3 vs. NL/InV2 (Figures 2A,B). These results may be due to the high affinity of the anti-Vpr antibody used. Taken all together, our results show that the nucleotide sequences of SA1D2prox and *vif*-coding region affect the expression levels of *vif*/*vpr* and Vif/Vpr in the context of proviral genome. Although most of our analyses were done in 293T cells, it has been demonstrated that the HIV-1 splicing pattern is essentially the same in infected lymphocytic CEMx174 cells and in transfected 293T cells (Emery et al., 2017).

## A3G-Degrading Activity of Chimeric Virus Clones InpInV, NL/InV1, and NL/InV3

Most conspicuous observation in Figure 2B was that clones InpInV, NL/InV1, and NL/InV3 express Vif remarkably more poorly than the others. We thus asked how these virus clones with such a low Vif expression level are functionally active against A3G. To monitor the A3G-degrading activity, 293T cells were co-transfected with virus clones and a flag-tagged A3G expression vector, and the A3G level within cells was determined by Western immunoblot assays as previously described (Yamashita et al., 2008, 2010). Expression levels of Vif and Gag-precursor were also determined to confirm the validity of the experiment. As shown in Figure 2C, compared with positive (NL4-3 and Indie) and negative (NL-∆Vif) controls, the three clones were found to degrade A3G to various degrees. While clearly low relative to NL4-3 and Indie, InpInV and NL/InV1 exhibited distinct A3G-degrading activity. NL/InV1 expressed a slightly lower level of Vif relative to InpInV, but showed slightly higher A3G-degrading activity. Finally, compared with NL-∆Vif, even NL/InV3 with the lowest ability displayed the A3G-degrading activity at an appreciable level.

## Growth Properties in Weakly A3G-Positive and Highly A3G-Positive Cell Lines of Chimeric Virus Clones That Express Various Levels of Vif

Changes in the Vif expression level affect HIV-1 growth in A3G-expressing cells (Mandal et al., 2009; Widera et al., 2013, 2014; Nomaguchi et al., 2016). We therefore analyzed the association between the altered Vif expression levels and the replication abilities. Viruses prepared from transfected 293T cells were inoculated into weakly A3G-positive CEM-SS cells, which express A3G below virus-restrictive level and highly A3G-positive H9 cells (Nomaguchi et al., 2016). As shown in Figure 2D, NL4-3 and all chimeric viruses grew well in CEM-SS cells, demonstrating no fundamental defects in the viruses tested. In contrast, various growth phenotypes were observed in H9 cells. In the first group of the three chimeric viruses (Figure 1D), while InpNLV exhibited similar growth potential to NL4-3, NLpInV and InpInV similarly grew more slowly than NL4-3. Thus, although the *vif*/Vif expression levels of InpNLV and NLpInV were estimated to be similar, their growth kinetics were different. However, we noted that NLpInV expressed more Vpr than InpNLV (Figure 2B). Considering inverse correlation between expression of the two proteins, it is conceivable that NLpInV may actually express a lower level of Vif relative to InpNLV, which could not be detected by the *vif*/Vif assays (Figures 2A,B). Another important point to be mentioned here is that, while the *vif*/Vif levels of InpInV were reduced relative to those of NLpInV (Figures 2A,B), the two clones grew similarly. In this regard, we previously found that the *vif* expression level in a certain range (0.16 to 0.47 relative to NL4-3) is sufficient to maintain wild-type growth ability (Nomaguchi et al., 2016). This may explain, at least in part, why the growth kinetics of the two clones were similar. In the second group of the four virus clones that carry chimeric *vif* sequences (Figure 1D), NL/InV1 and NL/InV3 showed a very attenuated growth phenotype in highly A3G-positive H9 cells. However, as compared with the low Vif-expressing InpInV, the decrease in replication ability of NL/InV1 and NL/InV3 was too drastic. It is conceivable that, for chimeric Vifs of NL/InV1 and NL/InV3, the alteration of functional structure in addition to their expression levels may severely negatively affect their anti-A3G activity. Indeed, consistent with the results in Figure 2C, NL/InV3 grew much more poorly than InpInV in H9 cells. Regarding NL/InV1, however, its inability to grow in H9 cells cannot be explained only by the defective anti-A3G activity. NL/InV1 showed a slightly higher A3G-degrading activity than InpInV (Figure 2C). Upon careful examination of the growth potentials of various chimeric clones, we noticed that, in CEM-SS cells, NL/InV1 grew more poorly than InpInV and also NL/InV3 (Figure 2D). This result was certainly reproduced in the two independently performed infection experiments (Figure 2E), and suggested that some activity of NL/InV1, other than the Vif activity, may be weaker than that of the other two chimeric clones. This possibility remains to be experimentally confirmed. Of note, nucleotide variations and/or spontaneous mutations in the central region of HIV-1 genome can affect the viral replication potential in a Vif-independent manner (our unpublished data).

In conclusion, our results here clearly show that nucleotide sequences of SA1D2prox and *vif* influence the Vif expression level to a virologically significant extent. While a certain level of the expression/function of Vif is a prerequisite for HIV-1 replication, it is possible that the level for Vpr expression, inversely correlated with Vif expression, may influence viral replication in some cell type. Vpr has been reported to exert its function mainly in the myeloid cell lineage (Fujita et al., 2010; Guenzel et al., 2014; González, 2017; Nodder and Gummuluru, 2019).

## Concluding Remarks

In this work, we have summarized and studied the effect of SA1D2prox and *vif*-coding sequences on the Vif expression level and virus replication ability. By utilizing sequences of both regions from HIV-1 subtypes B (NL4-3) and C (Indie) viruses with different anti-A3G activity, we generated chimeric proviral clones with the backbone of NL4-3 (Figure 1). Together with our previous reports (Nomaguchi et al., 2014, 2016, 2017), our results suggest that viral nucleotide sequences of both SA1D2prox and *vif* coding-region contribute to determining Vif expression level and consequently affecting HIV-1 replication (Figure 2). Interestingly, when natural Vif variants were expressed by a certain expression vector, their expression levels were not uniform (Simon et al., 2005; Ooms et al., 2013).

Many SREs have been identified around SAs/SDs including those within SA1D2prox and *vif*-coding sequence (Caputi, 2011; Karn and Stoltzfus, 2012; Sertznig et al., 2018). Since mutating and evolving activity under the host’s environments are characteristic of HIV-1, nucleotide sequences in these elements and/or in unknown SREs can often be changed. It is not unreasonable to assume that such natural variations may modify splicing regulations, and thus may alter the production of HIV-1 mRNAs and subsequent expression of viral proteins. Some of HIV-1 proviral clones that were generated by global synonymous mutagenesis exhibited replication defects caused by splicing perturbations, indicating the importance of the nucleotide sequence involved in splicing regulation (Takata et al., 2018). In individuals infected with HIV-1, Vif can alter its expression level and/or counteracting activity against A3s by naturally occurring mutations/variations. It would be advantageous for HIV-1 survival to maintain a certain level of antagonism achieved *via* the expression level and anti-A3 activity of Vif. Further analyses of the effect of natural non-synonymous and synonymous variations, found widely among HIV-1 strains, on the gene expression process including the splicing regulation would provide vital insights into the association between nucleotide changes and HIV-1 replication.

## Data Availability Statement

The datasets generated for this study are available on request to the corresponding author.

## Author Contributions

MN designed the research project. ND, TK, and MN performed the experiments. ND, TK, MN, and AA discussed the results. MN and AA wrote the manuscript. All authors approved its submission.

### Conflict of Interest

The authors declare that the research was conducted in the absence of any commercial or financial relationships that could be construed as a potential conflict of interest.

## Footnotes

^1^https://www.hiv.lanl.gov
